# Reduction in Heart Rate Variability with Traffic and Air Pollution in Patients with Coronary Artery Disease

**DOI:** 10.1289/ehp.0901003

**Published:** 2009-11-18

**Authors:** Antonella Zanobetti, Diane R. Gold, Peter H. Stone, Helen H. Suh, Joel Schwartz, Brent A. Coull, Frank E. Speizer

**Affiliations:** 1 Department of Environmental Health, Harvard School of Public Health, Boston, Massachusetts, USA; 2 Channing Laboratory, Brigham and Women’s Hospital, Department of Medicine, Harvard Medical School, Boston, Massachusetts, USA; 3 Cardiovascular Division, Brigham and Women’s Hospital, Harvard Medical School, Boston, Massachusetts, USA; 4 Environmental Statistics Program, Department of Biostatistics, Harvard School of Public Health, Boston, Massachusetts, USA

**Keywords:** air pollution, cardiac event, heart rate variability, myocardial infarction, traffic

## Abstract

**Introduction:**

Ambient particulate pollution and traffic have been linked to myocardial infarction and cardiac death risk. Possible mechanisms include autonomic cardiac dysfunction.

**Methods:**

In a repeated-measures study of 46 patients 43–75 years of age, we investigated associations of central-site ambient particulate pollution, including black carbon (BC) (a marker for regional and local traffic), and report of traffic exposure with changes in half-hourly averaged heart rate variability (HRV), a marker of autonomic function measured by 24-hr Holter electrocardiogram monitoring. Each patient was observed up to four times within 1 year after a percutaneous intervention for myocardial infarction, acute coronary syndrome without infarction, or stable coronary artery disease (4,955 half-hour observations). For each half-hour period, diary data defined whether the patient was home or not home, or in traffic.

**Results:**

A decrease in high frequency (HF; an HRV marker of vagal tone) of −16.4% [95% confidence interval (CI), −20.7 to −11.8%] was associated with an interquartile range of 0.3-μg/m^3^ increase in prior 5-day averaged ambient BC. Decreases in HF were independently associated both with the previous 2-hr averaged BC (−10.4%; 95% CI, −15.4 to −5.2%) and with being in traffic in the previous 2 hr (−38.5%; 95% CI, −57.4 to −11.1%). We also observed independent responses for particulate air matter with aerodynamic diameter ≤ 2.5 μm and for gases (ozone or nitrogen dioxide).

**Conclusion:**

After hospitalization for coronary artery disease, both particulate pollution and being in traffic, a marker of stress and pollution, were associated with decreased HRV.

Over the last 10 years epidemiologic studies have increasingly found higher daily morbidity and mortality associated with acute changes in ambient particulate matter (PM) exposures [Pope and Dockery 1999; U.S. Environmental Protection Agency (EPA) 2004]. These changes have been reported at concentrations at or near current ambient levels and have raised concerns about the adequacy of the National Air Quality Standards for PM in protecting human populations with an adequate margin of safety. Many of the early studies were criticized because mechanisms underlying the observed effects had not been elucidated at the time, particularly those related to cardiovascular morbidity and mortality. Recent toxicologic and human clinical studies have suggested a variety of PM-mediated mechanisms, including autonomic nervous system responses, particularly among patients with preexisting cardiovascular diseases (Chuang et al. 2007; Zanobetti and Schwartz 2007).

Heart rate variability (HRV), which is under the control of the autonomic nervous system, has been demonstrated to vary with air pollution (Delfino et al. 2005; Gold et al. 2000). Reduced HRV has been associated with increased risk of myocardial infarction in population studies (Tsuji et al. 1996) and has been considered a predictor of increased risk of mortality in patients with heart failure (Anonymous 1996). Although these studies did not assess the impact of acute changes in HRV, a recent study reported that ischemic events are preceded by decreases in high-frequency (HF) HRV in the hour before the event (Kop et al. 2001), and a decrease in HRV also has been reported to precede paroxysmal atrial fibrillation (Bettoni and Zimmermann 2002). Hence, short-term changes in HRV also may be risk factors for more serious consequences.

Local and regional traffic has been proposed as an important source of PM pollution that may increase cardiovascular risk, perhaps through triggering short-term autonomic changes. A recent German study demonstrated that exposure to local traffic within the previous hour was a trigger of myocardial infarction, but the mechanism through which this occurred was not determined (Peters et al. 2004). Traffic can be a source not only of PM pollution but also of stress, a known risk factor for adverse cardiac outcomes, and psychologic stress has been known to influence HRV and autonomic function (Anderson et al. 2007). In a study of traffic patrol troopers, traffic pollution inside the vehicle was demonstrated to increase HRV, systemic inflammation, and cardiac arrhythmias (Riediker et al. 2004). Most other studies have found reduction in HRV with pollution (Brook et al. 2004), but the directionality and clinical implications of the pollution-related cardiac autonomic changes likely vary with patient vulnerability.

With a specific focus on traffic pollution effects, we undertook a study of potentially susceptible patients with recent hospitalization for coronary artery disease to determine whether HRV was affected by ambient changes in PM pollution and a report of being in traffic.

## Materials and Methods

### Patient recruitment

The study design has been previously described (Chuang et al. 2008). Briefly, before they were discharged, we recruited a panel of patients with documented coronary artery disease from the greater Boston, Massachusetts, area (within Route 495, a 40-km radius of our central monitoring site) who had undergone percutaneous coronary intervention for an acute coronary syndrome (acute myocardial infarction or unstable angina pectoris) or for stable coronary artery disease with worsening symptoms, between October 1999 and January 2003. The presence of an acute myocardial infarction was defined as an elevation in creatine kinase (CK), CK-MB, or troponin I levels greater than three times the upper limit of normal in the setting of typical chest pain and electrocardiogram (ECG) changes. The diagnosis of prior myocardial infarction was extracted from the patients’ hospital record. We excluded those with atrial fibrillation and left bundle branch block because of the intent to evaluate HRV as well as ST-segment depression as outcomes. Coronary artery bypass graft surgery within the preceding 3 months was an exclusion criterion because the nonspecific ST and T-wave changes postoperatively would preclude accurate interpretation of new ST and T-wave changes. Patients with psychiatric illness or drug abuse problems were excluded because of compliance and reliability issues. Active cigarette smoking was an exclusion criterion at entry to the study, and no participant reported current smoking at the time of recruitment. Although four subjects reported recidivist smoking, each reported smoking at only one visit, and only one reported > 0–1 cigarettes per day at that visit (Table 1).

### Study protocol

The protocol included a home visit within 2–4 weeks after hospital discharge, followed by three additional follow-up visits at approximately 3-month intervals. At the first visit, a baseline screening questionnaire was administered regarding medications, pulmonary and cardiac symptoms, and smoking history.

Three-lead 24-hr Holter ECG monitoring (Marquette Seer Digital Recorder; Marquette Inc., Milwaukee, WI) was also performed with electrodes in modified V5 and aVF positions. During this monitoring, participants completed a standardized diary defining their activities and location for each half-hour period. For subsequent visits, participants were administered a brief questionnaire regarding cardiac and respiratory symptoms and medication use, and then received 24-hr Holter monitoring. We obtained written informed consent from the study subjects. The study design was reviewed and approved by the human subjects committees of the Brigham and Women’s Hospital and the Harvard School of Public Health.

Digitized Holter monitor recordings were analyzed using a Marquette MARS Workstation (GE Medical Systems, Milwaukee, WI). Tracings were initially reviewed for artifact by a trained technician. HRV was assessed using conventional time domain and frequency domain variables, measured using validated software (Anonymous 1996). Two patients on continuous positive airway pressure for sleep apnea were excluded from data analysis because of the potential influence of positive pressure on HRV outcomes. Time domain variables included the standard deviation of normal-to-normal heart beat intervals (SDNN) and the square root of the mean of the squared differences between adjacent normal RR intervals (r-MSSD). These values were averaged over 30-min clock time intervals to correspond with the resolution of the aerometric measures, which were collected over 30-min intervals. Frequency domain variables included HF measures and total power (TP). Both r-MSSD and HF were used to provide a measure of vagal activity, whereas SDNN and TP were used to provide a measure of sympathovagal activity. To our knowledge there are no validated criteria for half-hour HRV measures to define outliers. We therefore chose to use three times the median of the absolute differences as the cutoff for outliers. This resulted in a loss of approximately 13% of each of the HRV measures used in the analysis.

Information about diabetes diagnosis was abstracted from the medical records.

From diary data, the location of each participant was determined for each half-hour period, with location noted as home or not home, and in traffic defined as driving or riding in a car or riding a bus, subway, or train. For each 30-min interval, we classified each subject as in traffic in the previous 2 hr, not in traffic in the previous 2 hr, or in traffic part of the previous 2 hr. We similarly classified the subjects in relation to being home.

Analyses examined the association between HRV measures and both lagged and accumulated averages of ambient exposures to fine PM [≤ 2.5 μm in aerodynamic diameter (PM_2.5_)] and black carbon (BC) for periods ranging between 30 min to a few hours and up to 5 days preceding the HRV measures.

### Aerometrics

Ambient PM concentrations were measured at a central monitoring site located on the roof of Countway Library, Harvard Medical School, in downtown Boston for the years 1999 to 2003. The median distance of participant homes from the central site monitoring station was 17.6 km. Ambient measures included continuous PM_2.5_ concentrations measured using a tapered element oscillation microbalance (TEOM) (model 1400A; Rupprecht and Pastashnick, East Greenbush, NY) with a 2.5-μm (PM_2.5_) cut point. Ambient BC was measured using an aethalometer (model 8021; McGee Scientific, Berkeley, CA).

Hourly measurements of carbon monoxide (CO), ozone (O_3_), nitrogen dioxide (NO_2_), and sulfur dioxide (SO_2_) were obtained by taking the mean of site values for each gas from state monitoring sites in Boston: five sites for NO_2_ and SO_2_, four sites for CO, and three sites for O_3_. PM_2.5_ and BC and the gases concentrations were summarized in half-hour intervals and as running averages from 0.5 hr up to and including 120 hr (5-day moving averages).

Indoor PM_2.5_ was measured inside the homes of participants continuously using a TEOM with each measurement integrated over periods of half-hour, whereas indoor BC was measured using the aethalometer. BC was measured also outside the participants’ homes (called outdoor BC from now on). Hourly temperature was obtained from the National Weather Service first-order station at Logan Airport.

### Statistical analysis

We analyzed the association of HRV with air pollution using generalized additive fixed-effect regression models—that is, we included an indicator variable for each subject. This takes into account the effect of the within-subject similarities due to repeated measurements within each subject and removes the potential for confounding of subject-specific (time-invariant) factors (Hastie and Tibshirani 1990). In addition to controlling for each subject, the models also controlled for day of the week with indicator variables; for traffic; and for average heart rate, hour of the day, date, and mean temperature with penalized splines with 4, 6, 12, and 4 degrees of freedom (df), respectively. Results were not sensitive to changing the number of degrees of freedom for temperature and seasonality. Relative humidity was not a significant predictor.

Traffic was defined as a categorical variable as being in traffic in the previous 2 hr, not in traffic in the previous 2 hr, or in traffic part of the previous 2 hr. To eliminate concurvity between the penalized splines of date and temperature, we first regressed temperature against a penalized spline of date. We used the residuals from this model to control for temperature in our model for HRV. The outcome variables were log-transformed to meet normality assumptions. We assessed the relationship between ambient pollutant and HRV overall and for selected repeated time periods after acute hospitalization. The HRV measurements were analyzed with and without heart rate adjustment. To take into account correlation between adjacent half-hours, we used 6 df in the smoothing function of hour of the day. We checked the assumption of linear dose–response relationships used in the analyses. The analyses were performed using the statistical package R (R Development Core Team 2009).

In secondary analyses we assessed the effect modification by including an interaction term, with discharge diagnoses and history of diabetes. We also examined whether the previous 2-hr associations varied according to whether a subject was home for the previous 2 hr, not home during this time, or home only part of the 2 hr.

Results are presented as percent change scaling the PM_2.5_ effects and BC effects to the 25th to 75th interquartile range (IQR) for each pollutant at each averaging time period.

## Results

We enrolled 46 subjects, whom we studied in their homes on up to four occasions during the course of 1 year after their index event (Table 1). The subjects, all of whom had documented coronary artery disease, ranged in age from 43 to 75 years, approximately 80% were males, and all but three were Caucasian. Eighteen (39%) of the subjects experienced an acute myocardial infarction as part of the index hospitalization. Of the remaining 28 without an acute myocardial infarction, 18 (64%) had a history of previous myocardial infarction. Almost all participants were on statins, aspirin, and beta blockers. Approximately half of the patients (24) also were treated with angiotensin-converting enzyme inhibitors, with all other drugs being used by < 15% of the subjects. Participants with more than one follow-up visit were on average younger than participants with only one visit (57 vs. 62 years old) and were less likely to have ever had a myocardial infarction, although this difference was not statistically significant (Table 1). For the 46 subjects, we obtained 125 separate 24-hr ECG Holter recording sessions, with 4,955 half-hour observations. We had 3,179 observations for indoor PM_2.5_, 3,365 for indoor BC, and 2,508 for outdoor BC.

Ambient pollution levels were relatively low, with PM_2.5_ and gases never exceeded the current or proposed National Air Quality Standards (U.S. EPA 2004). However, the distribution of both ambient PM_2.5_ and BC concentrations during the study periods exhibited substantial variability, as evidenced by IQRs corresponding to an approximately 2.5-fold difference in concentrations. Pollutant variability was greatest for the shorter exposure periods (up to 4 hr). For the longer 3–5 day average concentrations, IQRs differed by 1- to 2-fold (Table 2). When assessing indoor pollution, indoor PM_2.5_ concentrations were slightly lower compared with the ambient, whereas the indoor BC concentrations were half those observed for the ambient BC. Ten subjects for a total of 17 of 125 visits reported very occasional smoking by other adults in the home “since we last saw you.” The mean levels for either BC or PM were slightly but not significantly higher for observations when smoking was reported versus when it was not (14 vs. 11 μg/m^3^ for PM half-hourly average).

### Ambient pollution and HRV

**Figure 1:**
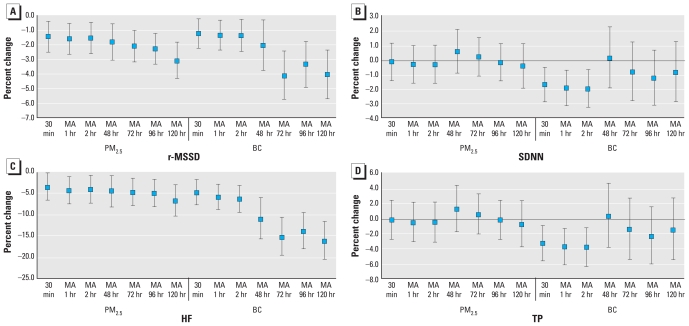
Percent change (95% CI) in r-MSSD (msec; *A*), SDNN (msec; *B*), HF (msec^2^; *C*), and TP (msec^2^; *D*) associated with different averaging times of PM_2.5_ and BC exposure. MA, moving average. PM_2.5_ and BC effects are scaled to their IQR.

Using ambient concentrations as the exposure measure, we found significant negative associations between both r-MSSD and HF and both PM_2.5_ and BC for all averaging periods. Overall, we observed a tendency for decreases to be larger with longer averaging times (Figure 1). For example, for PM_2.5_, at 1 hr for an IQR of 8.2 μg/m^3^ compared with the 5-day IQR average of 4.8 μg/m^3^, the decrease in r-MSSD was −1.47% [95% confidence interval (CI), −2.53 to −0.4%] versus −3.1% (95% CI, −4.35 to −1.85%). Effects of PM pollutants on percent change for HF were greater in magnitude than for r-MSSD across all the time periods examined (Figure 1), with a decrease in HF of −4.4% (95% CI, −7.6 to −1.15%) for an IQR increase in PM_2.5_ the previous hour and a decrease of −6.9% (95% CI, −10.5 to −3.1%) for an IQR increase in the 5-day moving average of PM_2.5_. The effects were even larger in association with BC with a decrease in HF of −16.4% (95% CI, −20.7 to −11.8%) for an IQR of 0.3-μg/m^3^ increase in BC for the 5-day moving average. In contrast, for all subjects taken together, the associations noted over the same time periods for SDNN and TP were smaller and were significant only in association with BC at short lags (Figure 1).

Previously we reported that those with diabetes had greater ST-segment depression with air pollution exposures (Chuang et al. 2008). Similarly, although we saw effects of pollution on HRV for people with and without diabetes by means of an interaction term, those with diabetes exhibited the largest response to pollution. For example, for a 3-day moving average of PM_2.5_, the decrease in r-MSSD was −0.9% (95% CI, −2.1 to 0.3%) in those without diabetes versus −9.5% (95% CI, −12.5 to −6.5%) in subjects with diabetes. For a 3-day moving average of BC, the decrease in r-MSSD was −3.2% (95% CI, −5.0 to −1.3%) in those without diabetes and −9.1% (95% CI, −13.2 to −4.9%) in those with diabetes. However, in contrast to our report on the outcome of ST-segment depression (Chuang et al. 2008), those without myocardial infarction but with unstable angina pectoris or worsening stable coronary artery disease showed a stronger effect of PM on HRV than did those with a discharge diagnosis of myocardial infarction. For a 3-day moving average of PM_2.5_, the decrease in r-MSSD was −2.9% (95% CI, −4.3 to −1.6%) for those without myocardial infarction but with unstable angina pectoris or worsening stable coronary artery disease versus 0.3% (95% CI, −1.8 to 2.4%) for those with myocardial infarction. For a 3-day moving average of BC, the decrease in r-MSSD was −5.9% (95% CI, −7.9 to −3.9%) for those without myocardial infarction but with unstable angina pectoris or worsening stable coronary artery disease, and −0.4% (95% CI, −3.1 to 2.4%) for those with a myocardial infarction. The interaction for diagnosis of diabetes and myocardial infarction was always significant, with a *p*-value < 0.001. Because > 90% of our patients were consistently taking statins and beta blockers throughout the study, we could not evaluate whether these medications were protective against pollution effects.

### Indoor pollution and HRV

**Figure 2:**
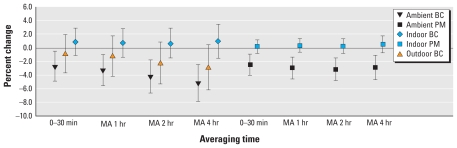
Percent change (95% CI) in r-MSSD (msec) associated with different averaging times of indoor PM_2.5_ and BC exposure. MA, moving average. PM_2.5_ and BC effects are scaled to 10 and 1 μg/m^3^, respectively.

Table 3 and Figure 2 present the effects of indoor compared with outdoor pollution measures on HRV, and the effects of ambient pollution when a subject was at home versus away from home. Because there were more ambient than indoor observations, for comparability of results in Figure 2, we present the indoor, outdoor, and ambient results from the data set with the same number of observations for both indoor and ambient. Table 3 focuses on the more immediate effects of pollution, because we wanted to evaluate cumulative pollution exposures during a period comparable with time spent in traffic (2 hr), or to time spent at home, given that most participants left the house for portions of the day.

The percent changes in Figure 2 and Table 3 are scaled to 10 μg/m^3^ for PM_2.5_ and to 1 μg/m^3^ for BC. We found no significant effects of pollution on HRV when the subjects were home, either for ambient or for indoor measured pollution. When subjects were home part of the time, the magnitude of the association of ambient pollution with reduced HRV was larger than when they were home all of the time, but smaller than when they were away from home all of the time. For example, when the patients were away from home during the previous 2 hr, we found a 7.9% (95% CI, −10.3 to −5.3%) decrease in r-MSSD for a 10-μg/m^3^ increase in PM_2.5_ and a 6.9% (95% CI, −10.1 to −3.6%) decrease for a 1-μg/m^3^ increase in BC, compared with a 0.4% (95% CI, −1.3 to 2.1%) decrease and a 0.45% (95% CI, −1.8 to 2.8%) decrease, respectively, when the patients were home. We also found a 14.8% (95% CI, −21.6 to −7.4%) decrease in HF for a 10-μg/m^3^ increase in PM_2.5_ and a 17.4% (95% CI, −25.9 to −8%) decrease for a 1-μg/m^3^ increase in BC when the patients were away from home during the previous 2 hr, compared with a 1.6% (95% CI, −6.6 to 3.6%) decrease and a 5.2% (95% CI, −11.7 to 1.7%) decrease, respectively, when the patients were home.

### Traffic and HRV

We defined traffic as driving or riding in a car or riding a bus, subway, or train; cars were the mode of transport for 90% of the observations for time in traffic. Traffic exposure in the previous 2 hr had the largest effect on reduction of HRV, which was independent of the effects of ambient central site–measured BC (Table 3). Traffic was associated with a 15% decrease in r-MSSD and a 39% decrease in HF. We found a dose–response relationship: When the patients were in traffic only part of the previous 2 hr, traffic exposure was associated with a 3% decrease in r-MSSD and a 5% decrease in HF.

### Ambient gases and HRV

**Figure 3:**
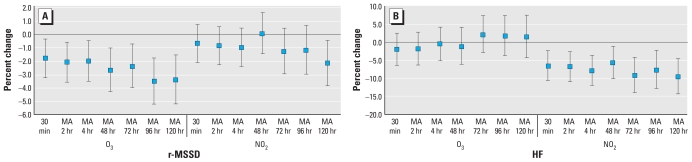
Percent change (95% CI) in r-MSSD (msec; *A*) and HF (msec^2^; *B*) associated with different averaging times of O_3_ and NO_2_ exposure. MA, moving average. O_3_ and NO_2_ effects are scaled to their IQR.

We found decreases in r-MSSD for all averaging time of O_3_ with a 2.1% (95% CI, −3.5 to −0.6%) decrease for an IQR increase of 19 ppb mean O_3_ in the previous 2 hr and a 3.4% (95% CI, −5.2 to −1.5%) decrease for an IQR increase of 13 ppb in the 5-day moving average of O_3_ (Figure 3). HF was not associated with O_3_ but was associated with NO_2_ for all averaging times. The association between NO_2_ and HF was relatively large and consistent across all averaging times, with a decrease of 6.7% (95% CI, −10.8 to −2.5%) for an IQR increase of 0.011 ppm in the 2-hr average NO_2_, and a decrease of 9.4% (95% CI, −14.1 to −4.4%) for an IQR increase of 0.006 ppm in the 5-day average of NO_2_.

The correlation between the gases and PM_2.5_ and BC was only modest, with a correlation for the 72-hr average PM_2.5_ of 0.46 with NO_2_ and 0.29 with O_3_; the correlation of BC for the 72-hr average was 0.46 with NO_2_ and −0.08 with O_3_.

We tested two-pollutant models using each of the gas and PM measures together (Table 4). For both r-MSSD and HF and for both PM_2.5_ and BC, the addition of O_3_ or NO_2_ to the model did not significantly alter the effects seen in the single-pollutant models. Both O_3_ and PM_2.5_ had independent effects on reduced HF.

## Discussion

These results suggest that short-term traffic exposure and ambient exposure to air pollution are associated with significant reductions in HRV in free-living patients with underlying heart disease, and that recent traffic exposure can cause reductions in HRV as great as 39% in patients 2–6 weeks after hospitalization for coronary artery disease. As in previous studies (Adar et al. 2007b; Gold et al. 2000; Schwartz et al. 2005), we found stronger autonomic dysfunction for HRV measures that represent vagal tone (r-MSSD and HF) than for HRV measures representing overall autonomic responses or sympathetic tone (SDNN and TP). We found associations for both at shorter (2 hr) as well as at longer averaging times (up to 5 days). We observed the greatest effects of PM and BC for longer cumulative averages.

We may not have found an influence of indoor air pollution on HRV because the indoor levels were lower, with few indoor sources of indoor pollution. However, in this same cohort, we did find an effect of both outdoor and indoor BC on increases in T-wave alternans (Zanobetti et al. 2009), an electrophysiologic measure of alternating repolarization waveform indicative of instabilities in cardiac membrane voltage and disruptions in intracellular calcium cycling dynamics. In our cohort, the T-wave alternans was more influenced by short-term cumulative averages of pollution and may be linked to pollution exposure through pathways different from those of HRV. Pollution may influence HRV through direct stimulation of pulmonary reflexes, leading to cardiac or systemic autonomic dysfunction, or through pulmonary/systemic inflammation or oxidative stress, which may lead to autonomic dysfunction (Adamkiewicz et al. 2004; Adar et al. 2007a; Chuang et al. 2007; Park et al. 2005; Peters et al. 2006).

The associations of ambient (central site–measured) BC with our outcomes were uniformly stronger than the associations of BC outside the home with our outcomes (Figure 2). It is possible that ambient central site measures of BC had stronger and larger associations with reduced HRV than did outdoor “outside the home” measures because our ambient measures provide a better measure of regional average exposure during the times when our participants were away from home. The association of reduced HRV in single-pollutant models with only NO_2_, which can be considered another marker of traffic in addition to BC, provides further support for the effects of pollution of traffic origins. The data also suggest that mixtures of pollutions may influence autonomic cardiac tone, in that both PM pollution and O_3_ had independent associations with reduced HRV in two-pollutant models.

Our results are consistent with the previous literature on potentially high-risk patients, defined as elderly subjects (with or without documented coronary heart disease) (Creason et al. 2001; Gold et al. 2000; Holguin et al. 2003; Liao et al. 2004; Pope et al. 2004). Others have studied pollution responses in patients with stable coronary heart disease (Lipsett et al. 2006; Timonen et al. 2006), whereas we studied our subjects 2–6 weeks after hospitalization for myocardial infarction, unstable angina pectoris, or worsening stable coronary artery disease. Autonomic dysfunction can precede more serious cardiac electrophysiologic disturbances in vulnerable patients (Lombardi et al. 2000; Monmeneu et al. 2001; Shusterman et al. 2000). HRV is a predictor of long-term cardiac mortality (de Bruyne et al. 1999), although the debate continues as to whether it is a marker or cause of increased cardiac risk. Previously we have described pollution-associated ST-segment depression (Chuang et al. 2008) and increases in T-wave alternans (Zanobetti et al. 2009) in the subjects participating in our study; the finding of traffic- and pollution-associated autonomic dysfunction adds to the evidence that pollution is increasing cardiac risk in this vulnerable population.

Within this population that was vulnerable on the basis of coronary artery disease, people with diabetes were even more vulnerable. This adds to cumulative evidence that type 2 diabetes, which is accompanied by a chronic inflammatory state and by chronic autonomic dysfunction, is a source of vulnerability to pollution (Dubowsky et al. 2006; O’Neill et al. 2005, 2007; Zanobetti and Schwartz 2001, 2002).

The major limitation of this study, besides the relatively small number of study participants, was that we did not study all subjects on all occasions. In addition, although the strongest associations of HRV related to the previous 3 days’ ambient PM exposures, 72-hr integrated indoor or personal exposure measurements were not available for comparison hours. More minor limitations relate to the self-report of medication use, without substantial documentation of compliance. The finding that increasing length of averaging times was associated with increasing effects could relate either to increasing biologic cumulative effects over time or to less measurement error over longer averaging periods. However, in this study we had better estimates of exposure than in most air pollution health effects studies using administrative data only. Although it is possible that our results could reflect time spent indoors at home immediately after hospitalization or time spent sleeping, we did not find that either of these factors modified the effects of indoor pollution on HRV. For the few subjects with environmental tobacco smoke (ETS) exposure in the home “since we last saw you,” it is possible that the indoor PM levels were, in part, due to ETS rather than ambient pollution penetrating indoors, cooking, or cleaning. However we found no association of indoor PM levels with reduced HRV with or without the observations where there was ETS in the home “since we last saw you.”

Although it is possible that being in traffic represents a combination of exposures, we have confidence that the effects of traffic are not a result of chance or the specific vulnerability of a few subjects. Being in traffic for either part or all of the 2 hr before and including HRV measurement was associated with reduced HRV. Although we have only 27 observations for 6 subjects who were in traffic for a full 2 hr, 40 subjects contributed 315 observations when they were in traffic for part of the previous 2 hr (at least one half-hour). It is, however, possible that being in traffic is a measure of a combination of factors with adverse effects. Studies have suggested that stress can trigger adverse cardiac events, including myocardial infarction or arrhythmias (Albert et al. 2007). A limitation of our study is that we did not measure stress while in traffic and therefore cannot disentangle the combination of pollution and stress exposures that “being in traffic” represents. Nevertheless, in the models including “being in traffic” and in the models stratified by being indoors and not in traffic, the independent associations of measured pollution with reduced HRV are not likely to be confounded by the stress of being in traffic. We did not have details on pollution levels in the cars of our study participants. Ventilation in vehicles can vary widely. It would be useful for future studies to evaluate whether reduction of in-vehicle pollution improves HRV and other indicators of cardiac risk in vulnerable populations.

This study provides further evidence that exposure to ambient pollution to both PM_2.5_ and BC alters autonomic function, particularly among subjects with a history of previous myocardial injury. Traffic exposure, which likely represents a complex combination of local pollution exposure and stress, independently adds to risk incurred by the background of regional pollution. Mixtures with gases such as O_3_ may add to the risk of PM pollution exposure. Susceptible populations such as individuals with coronary artery disease should be considered when setting national policies and regulations to control levels of PM air pollution and traffic.

## Figures and Tables

**Table 1:** Participant characteristics (*n* = 46) followed up after an acute coronary event.

Characteristic	Median (range)	*n* (%)
Age (years)	57 (43–75)	
r-MSSD (msec)	25.3 (6.8–83.5)	
SDNN (msec)	66.8 (11.7–147.7)	
HF (msec^2^)	90.5 (0.2–1,235)	
TP (msec^2^)	4,381 (125.1–21,650)	

Sex		
Male		37 (80.4)
Female		9 (19.6)

No. of visits		
1		45 (36.0)
2		34 (27.2)
3		24 (19.2)
4		22 (17.6)
Total		125 (100)

Diagnosis		
Myocardial infarction current		18 (39.1)
Coronary artery disease, not myocardial infarction		28 (60.9)

Ever used medication		
Statin		42 (91.3)
Aspirin		41 (89.1)
Beta blocker		42 (91.3)
Calcium channel blocker		5 (10.9)
Angiotensin-converting enzyme inhibitors		24 (52.2)
Asthma: theophylline and beta antagonist		2 (4.3)
Digoxin		2 (4.3)

Diabetes		
No		35 (76.1)
Yes		11 (23.9)

Cigarette smoking		
Never		15 (32.6)
Former		27 (58.7)
Current		4 (8.7)

Ethnic group		
White		43 (93.5)
Black		1 (2.2)
Asian and other		2 (4.3)

**Table 2:** Distribution of air pollution and weather variables for all subjects (*n* = 46).

	Percentile					
Variable	5th	25th	50th	75th	95th	IQR
Mean temperature (°C)	−6.1	3.3	10	18.9	27.8	

Ambient pollution						
PM_2.5_ (μg/m^3^)						
30 min	1.96	5.44	9.06	13.66	25.54	8.34
2-hr mean	2.54	5.65	8.92	13.58	25.07	7.93
72-hr mean	4.72	7.16	9.93	12.65	19.31	5.50
120-hr mean	5.48	7.6	9.54	12.34	15.95	4.83

BC (μg/m^3^)						
30 min	0.2	0.41	0.69	1.04	1.9	0.62
2-hr mean	0.22	0.42	0.70	1.03	1.83	0.61
72-hr mean	0.38	0.59	0.80	1.07	1.41	0.48
120-hr mean	0.43	0.59	0.74	0.93	1.16	0.34

NO_2_ (ppm)						
30 min	0.011	0.017	0.021	0.027	0.036	0.011
2-hr mean	0.011	0.017	0.021	0.027	0.036	0.010
72-hr mean	0.015	0.018	0.021	0.025	0.031	0.007
120-hr mean	0.015	0.018	0.021	0.024	0.029	0.006

O_3_ (ppb)						
30 min	2.04	11.36	20.73	30.33	53.96	18.97
2-hr mean	2.29	11.34	20.47	30.08	54.29	18.74
72-hr mean	7.95	15.71	21.87	28.33	42.33	12.62
120-hr mean	9.66	16.72	22.75	29.28	38.72	12.56

Indoor pollution						
PM_2.5_ (μg/m^3^)						
30 min	1.39	4.46	7.88	12.41	31.17	7.95
2-hr mean	1.84	4.68	8.16	12.56	31.98	7.88
4-hr mean	2.01	4.88	8.43	12.79	30.3	7.91

BC (μg/m^3^)						
30 min	0.1	0.21	0.38	0.62	1.17	0.41
2-hr mean	0.11	0.22	0.39	0.62	1.14	0.4
4-hr mean	0.11	0.22	0.4	0.63	1.12	0.41

Outdoor “at home” BC (μg/m^3^)						
30 min	0.11	0.25	0.47	0.78	1.42	0.52
2-hr mean	0.11	0.26	0.49	0.77	1.38	0.51
4-hr mean	0.12	0.28	0.49	0.78	1.29	0.5

**Table 3:** Percent change in r-MSSD and HF for ambient and indoor PM_2.5_ and BC: effects of control for traffic exposure on the association with ambient PM and effect modification.

		Percent change (95% CI)	
Exposure		r-MSSD	HF
Models including ambient PM_2.5_			
Model 1	2-hr mean ambient PM_2.5_	−2.0 (−3.3 to −0.6)	−5.2 (−9.2 to −1.1)
	In traffic, previous 2 hr	−15.2 (−24.8 to −4.4)	−39.2 (−58.0 to −12.0)
	In traffic, part of the previous 2 hr	−2.8 (−5.4 to −0.2)	−4.8 (−12.4 to 3.4)
Model 2	2-hr mean ambient PM_2.5_	−2.2 (−3.6 to −0.9)	−5.9 (−9.8 to −1.8)
Model 3	2-hr mean ambient PM_2.5_, not home	−7.9 (−10.3 to −5.3)	−14.8 (−21.6 to −7.4)
	2-hr mean ambient PM_2.5_, home	0.4 (−1.3 to 2.1)	−1.6 (−6.6 to 3.6)
	2-hr mean ambient PM_2.5_, home part of time	−4.0 (−7.0 to −0.9)	−9.3 (−17.8 to 0.0)

Models including indoor PM_2.5_ at home			
Model 4	30-min mean indoor PM_2.5_	0.2 (−0.8 to 1.3)	−0.8 (−4.0 to 2.5)
Model 5	2-hr mean indoor PM_2.5_	0.0 (−1.1 to 1.2)	−1.3 (−4.9 to 2.5)

Models including ambient BC			
Model 1	2-hr mean ambient BC	−2.2 (−4.0 to −0.4)	−10.4 (−15.4 to −5.2)
	In traffic, previous 2 hr	−15.7 (−25.2 to −5.0)	−38.5 (−57.4 to −11.1)
	In traffic, part of the previous 2 hr	−2.9 (−5.5 to −0.3)	−4.9 (−12.5 to 3.3)
Model 2	2-hr mean ambient BC	−2.5 (−4.3 to −0.7)	−11.1 (−15.9 to −5.9)
Model 3	2-hr mean ambient BC, not home	−6.9 (−10.1 to −3.6)	−17.4 (−25.9 to −8.0)
	2-hr mean ambient BC, home	0.4 (−1.8 to 2.8)	−5.2 (−11.7 to 1.7)
	2-hr mean ambient BC, home part of time	−6.5 (−10.2 to −2.6)	−22.2 (−31.5 to −11.6)

Models including indoor BC at home			
Model 4	30-min mean indoor BC	2.1 (0.0 to 4.2)	2.1 (−4.3 to 8.8)
Model 5	2-hr mean indoor BC	2.0 (−0.2 to 4.4)	2.3 (−4.7 to 9.8)

Each model evaluates air pollution using different metrics of exposure as listed in the table. All models adjust for subject and day of the week, average heart rate, hour of the day, date, and mean temperature as described in the text. For example, model 2 has a term for 2-hr mean ambient BC but does not include the three-level traffic variable. PM_2.5_ and BC effects are scaled to 10 and 1 μg/m^3^, respectively.

**Table 4:** Percent change in r-MSSD and in HF for the 72-hr mean in two-pollutant models.

	Percent change (95% CI)	
Variable (72-hr mean)	PM_2.5_	BC
r-MSSD		
NO_2_	1.16 (−0.97 to 3.34)	2.27 (0.00 to 4.59)
PM_2.5_ or BC	−2.32 (−3.41 to −1.21)	−5.53 (−7.71 to −3.29)
O_3_	−1.13 (−2.92 to 0.69)	−2.50 (−4.11 to −0.86)
PM_2.5_ or BC	−1.71 (−2.71 to −0.70)	−4.06 (−5.81 to −2.28)

HF		
NO_2_	−7.63 (−13.44 to −1.44)	1.06 (−5.61 to 8.21)
PM_2.5_ or BC	−2.38 (−6.49 to 1.91)	−15.36 (−20.99 to −9.32)
O_3_	6.89 (0.99 to 13.15)	1.81 (−3.33 to 7.23)
PM_2.5_ or BC	−7.09 (−10.65 to −3.39)	−14.72 (−19.22 to −9.97)

Pollutant effects are scaled to IQR.
